# Application of Improved Three-Dimensional Kernel Approach to Prediction of Protein Structural Class

**DOI:** 10.1155/2013/625403

**Published:** 2013-06-26

**Authors:** Xu Liu, Yuchao Zhang, Hua Yang, Lisheng Wang, Shuaibing Liu

**Affiliations:** ^1^School of Chemistry & Chemical Engineering, Guangxi University, Guangxi Province, Nanning 530004, China; ^2^State Key Laboratory of Medical Genomics, Institute of Health Sciences, Shanghai Institutes for Biological Sciences, Chinese Academy of Sciences, Shanghai Jiao Tong University School of Medicine, Shanghai 200240, China; ^3^Graduate School of the Chinese Academy of Sciences, Beijing 100049, China; ^4^College of Pharmacy, Guangxi University of Chinese Medicine, Nanning 530001, China

## Abstract

Kernel methods, such as kernel PCA, kernel PLS, and support vector machines, are widely known machine learning techniques in biology, medicine, chemistry, and material science. Based on nonlinear mapping and Coulomb function, two 3D kernel approaches were improved and applied to predictions of the four protein tertiary structural classes of domains (all-**α**, all-**β**, **α**/**β**, and **α** + **β**) and five membrane protein types with satisfactory results. In a benchmark test, the performances of improved 3D kernel approach were compared with those of neural networks, support vector machines, and ensemble algorithm. Demonstration through leave-one-out cross-validation on working datasets constructed by investigators indicated that new kernel approaches outperformed other predictors. It has not escaped our notice that 3D kernel approaches may hold a high potential for improving the quality in predicting the other protein features as well. Or at the very least, it will play a complementary role to many of the existing algorithms in this regard.

## 1. Introduction

Due to the rapid development of genome and protein science, the biological information has expanded dramatically. Therefore, it is very important and highly desirable for computers to manage, organize, and interpret the information. As a part of biochemistry, study of protein structure classes has become a hot topic, because of experimental and theoretical purposes. Artificial neural networks, support vector machines, kernel methods, and ensemble algorithms are widely known machine learning techniques in biology, medicine, chemistry, and material science [1–10]. In this work, two classification problems, protein's tertiary structure classes of domains and membrane protein types, were researched with some machine learning techniques. 

Several motifs pack together to form compact, local, and semiindependent units called domains. The details of proteins domains structures are extremely complicated and irregular. But their overall structural frames are simple, regular, and truly elegant [11–13]. Many protein domains often have similar or identical folding patterns even if they are quite different according to their sequences [14–16]. The overall 3D structure of the polypeptide chain is referred to as the protein's tertiary structure. Levitt and Chothia proposed to classify protein tertiary structures into the following four structural classes based on the secondary structural content of the domains. (1) All-*α*: it is formed essentially by *α*-helices. This class is dominated by small folds, many of which form a simple bundle with helices running up and down. (2) All-*β*: this class has a core composed of antiparallel *β*-sheets, usually two sheets pack against each other. (3)  *α*/*β*: this class contains both *α*-helices and *β*-strands that are largely interspersed in forming mainly parallel *β*-sheet; (4)  *α* + *β*: this class also contains both of the two secondary structure elements that, however, are largely segregated in forming mainly antiparallel *β*-sheets. 

This concept of structural class has ever since been widely used as an important attribute for characterizing the overall folding type of proteins domains. Lots of methods have been made to predict the structural classes based on the knowledge of protein sequences [17]. 

The research of membrane protein type is also important because of the special biological functions. The biomembrane usually contains some specific proteins and lipid components that enable it to perform its unique roles in the cell and organelle. 

Furthermore, several studies show that many membrane proteins are also the key targets of drug discovery, particularly membrane channel proteins [18–20]. Membrane proteins can be further classified into the five types [21–23]: (a) type A membrane protein is single-pass transmembrane protein which has an extracellular (or luminal) N-terminus and cytoplasmic C-terminus for a cell (or organelle) membrane; (b) type B membrane protein is single-pass transmembrane protein which has an extracellular (or luminal) C-terminus and cytoplasmic N-terminus for a cell (or organelle) membrane; (c) type C is multipass transmembrane protein: the polypeptide crosses the lipid bilayer multiple times; (d) type D membrane proteins are lipid chain-anchored membrane proteins: they are bound to the membrane by one or more covalently attached fatty acid chains or other types of lipid chains called prenyl groups; (e) type E is GPI-anchored membrane protein which is bound to the membrane by a glycosylphosphatidylinositol (GPI) anchor. 

**Figure 1:**
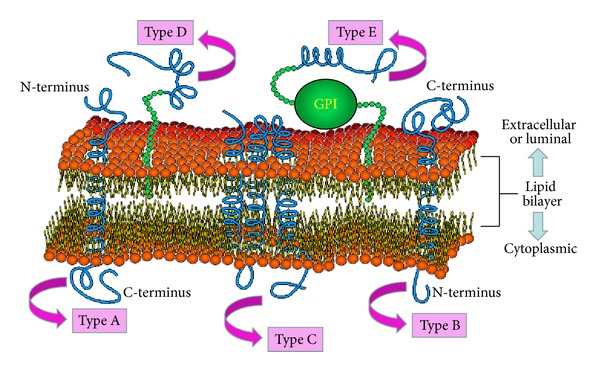
Five types of membrane proteins.

Researchers have applied classification algorithm to predict the types of membrane proteins based on their amino acid composition [24, 25].  Figure 1 shows the forms and the locations of different membrane proteins. 

The first goal of this paper is to illustrate the application of 3D kernel approach as a relatively new tool in proteins domains field for classification purposes. And the second goal is to show that the new approach can be applied to analysis of membrane protein types.

## 2. Materials and Methods

### 2.1. Kernel Function

Kernel function was originally a kind of functions used in integral operator research. However, Vapnik implemented this function in his newly invented SVMs method [26]. The use of kernel function makes SVMs able to treat nonlinear data processing problems by using linear algorithms. The basic idea of kernel function is to map the data **X** into a higher-dimensional feature space **F** via a nonlinear mapping Φ and then to do classification and regression in this space. There are four commonly used kernel functions:

 linear kernel
  (1) $$\begin{array}{c} K\left(x,y\right)=\langle x\cdot y\rangle +\theta . \end{array}$$
 polynomial kernel
  (2) $$\begin{array}{c} K\left(x,y\right)={\left(\langle x\cdot y\rangle +\theta \right)}^{d}. \end{array}$$
 Gaussian (RBF) kernel
  (3) $$\begin{array}{c} K\left(x,y\right)=\exp\left(\frac{-{||x-y||}^{2}}{\sigma^{2}}\right). \end{array}$$
 sigmoid kernel
  (4) $$\begin{array}{c} K\left(x,y\right)=\text{ tanh }\left(v\langle x\cdot y\rangle +r\right). \end{array}$$

The elegance of using kernel function lied in the fact that one can deal with feature spaces of arbitrary dimensionality without having to compute the map Φ(**x**). Any function that satisfies Mercer's condition can be used as kernel function.

### 2.2. Kernel PCA

Principal component analysis (PCA) is a versatile and easy-to-use multivariate mathematical-statistical method in multivariate data analysis and the extraction of maximal information [27, 28]. It is a linear transformation approach that compresses high-dimensional data with minimum loss of data information. PCA is performed in the original sample space, whereas kernel PCA (KPCA) applies kernel functions in the input space to achieve the same effect of the expensive nonlinear mapping. 

**Figure 2:**
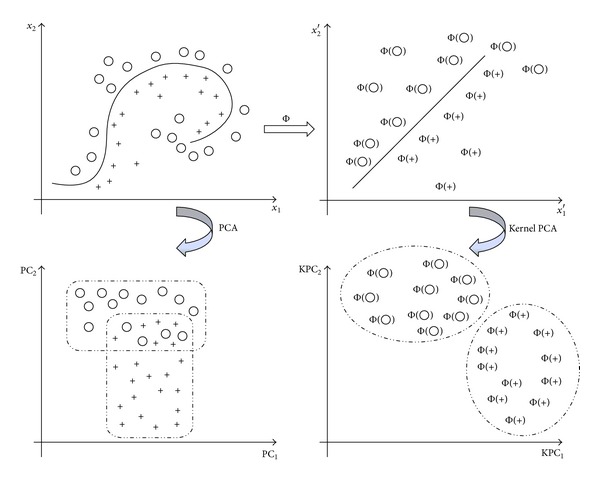
The mapping Φ embeds the data points in a feature space.

From Figure 2, it is found that the basic idea of KPCA is to map the original dataset into some higher dimensional feature space. In this complex space, PCA can be applied to establish a linear relationship which is nonlinear in the original input space [29, 30]. For the special case in which Φ(**x**) = **x**, KPCA is equivalent to linear PCA. From this viewpoint, KPCA can be regarded as a generalized version of linear PCA. 

For PCA, with data **X** = [*x*_1_, *x*_2_,…,*x*_*n*_]^*T*^ ∈ *R* ^*p*^, one can first compute the covariance matrix **C**:

(5) $$\begin{array}{c} C=\frac{1}{n}X^{T}X. \end{array}$$

A principal component **v** is computed by solving the following eigenvalue problem:

(6) $$\begin{array}{c} \lambda v=Cv=\frac{1}{n}X^{T}Xv. \end{array}$$

Thus, the eigenvectors can be written as (**α** = [*α*_1_,…,*α*_*n*_]^*T*^)

(7) $$\begin{array}{c} v=\sum_{i=1}^{n}\alpha_{i}x_{i}=X^{T}\alpha . \end{array}$$

Then, the eigen value problem can be represented by the following simple form:

(8) $$\begin{array}{c} \lambda \alpha =\frac{1}{n}K\alpha , \end{array}$$

where **K** = **X** **X** ^**T**^ ∈ *R* ^*n*×*n*^ is a linear kernel matrix. To derive KPCA, one firstly needs to map the data **X** into a feature space **F**  (i.e.,  **M** = [Φ(**x**_1_), Φ(**x**_2_),…,Φ(**x**_*n*_)]^*T*^). Hence, a nonlinear kernel matrix **K**  (**K** = **M** **M** ^**T**^ ∈ *R* ^*n*×*n*^) can be directly generated by means of specific kernel function ((1), (2), (3), and (4)). For extracting features of a new sample *x* with KPCA, one simply projects the mapped sample Φ(**x**) onto the first *k* projections **V**_*k*_,

(9) $$\begin{array}{c} V_{k}\cdot \Phi \left(x\right)=\sum_{i=1}^{n}\alpha_{i}^{k}\langle \Phi \left(x_{i}\right),\Phi \left(x\right)\rangle . \end{array}$$

KPCA is to map the original data (in the input space) with nonlinear features into kernel feature space in which the linear PCA algorithm is then performed. Therefore, KPCA, being suitable to describe the nonlinear structure of data set, can be regarded as a generalized version of linear PCA. 

### 2.3. GDA

Generalized discriminant analysis (GDA) is a method designed for nonlinear classification [31–33]. It is a nonlinear extension of linear discriminant analysis (LDA) based on a kernel function Φ which transforms the original space **X** to a new high dimensional feature space **F**. The within-class (or total) scatter (**W** ^Φ^) and between-class scatter (**B** ^Φ^) matrixes of the nonlinearly mapped data are as follows:

(10) $$\begin{array}{c} W^{\Phi }=\sum_{c=1}^{C}\sum_{x\in X_{c}}\Phi \left(x\right){\Phi \left(x\right)}^{T}, \end{array}$$

(11) $$\begin{array}{c} B^{\Phi }=\sum_{c=1}^{C}M_{c}m_{c}^{\Phi }{\left(m_{c}^{\Phi }\right)}^{T}. \end{array}$$

In (11), **m**_*c*_ is the mean of class **X**_*c*_ and *M*_*c*_ is the number of samples belonging to **X**_*c*_. The aim of the GDA is to find such projection matrix **U** ^Φ^ that maximizes the following Fisher criterion:

(12) $$\begin{array}{c} U_{\text{opt}}^{\Phi }=\arg\max\frac{\left|{\left(U^{\Phi }\right)}^{T}B^{\Phi }U^{\Phi }\right|}{\left|{\left(U^{\Phi }\right)}^{T}W^{\Phi }U^{\Phi }\right|}=\left[u_{1}^{\Phi },\ldots ,u_{N}^{\Phi }\right]. \end{array}$$

From the theory of reproducing kernels, any solution **u** ^Φ^ ∈ **F** must lie in the span of all training samples in **F**:

(13) $$\begin{array}{c} u^{\Phi }=\sum_{c=1}^{C}\sum_{i=1}^{M_{c}}\alpha_{ci}\Phi \left(x_{ci}\right), \end{array}$$

where *α*_*ci*_ are some real weights and *x*_*ci*_ is the *i*th sample of the class *c*. The solution is obtained by solving (**α** = [**α**_*c*_], *c* = 1,2,…, *C*; **α**_*c*_ = [**α**_*ci*_], *i* = 1,2,…, *M*_*c*_):

(14) $$\begin{array}{c} \lambda =\frac{\alpha^{T}KDK\alpha }{\alpha^{T}KK\alpha }. \end{array}$$

**K** is the *n* × *n* kernel matrix composed of the dot products of nonlinearly mapped data. And **D** = diag⁡(**D**_1_,…, **D**_*c*_), where **D**_*i*_ is a *n*_*i*_ × *n*_*i*_ matrix with entries all equal to 1/*n*_*i*_. 

### 2.4. New Improved 3D Kernel Approach: 3D KPCA and 3D GDA

Traditional KPCA and GDA are typical multivariate two-dimension statistical methods. In this work, KPCA and GDA are improved with three-dimensional projection and the concept of electric field intensity. 

Firstly, the data of training samples are projected onto three-dimensional space by KPCA or GDA algorithm with satisfactory classification effect. The three-dimensional coordinate axes are, respectively, the first kernel principal component, second kernel principal component, and third kernel principal component or the direction vectors of generalized discriminant analysis. 

Secondly, we need to estimate the class (unknown) of new projection points, such as membrane protein types of test sample data. There are two estimation methods in this work: K-Nearest Neighbor algorithm (KNN) [34] and class intensity model. 

KNN algorithm estimation: new projection point (test sample) is classified by a majority vote of its neighbors (training samples in kernel three-dimensional space).

Class intensity model estimation: the projection point of one training data can be considered as point charge. The species of charge is related to the class of sample. And the Electric Quantity of Point Charge (EQPC) is related to the number of samples (*n*_*C*_) which belongs to some class:

(15) $$\begin{array}{c} {\text{EQPC}}_{C}=\frac{1}{n_{C}}. \end{array}$$

The value of EQPC is negative related with the sample amount of same class. Based on the Coulomb law and formula of intensity of electric field, the Intensity of Electric Field of one Point (IEFP) in 3D space is

(16) $$\begin{array}{c} {\text{IEFP}}_{C}=\sum_{i=1}^{n_{C}}\frac{{\text{EQPC}}_{C}}{r_{i}^{2}}, \end{array}$$

where *r* is distance between point charge and the space point. 

**Figure 3:**
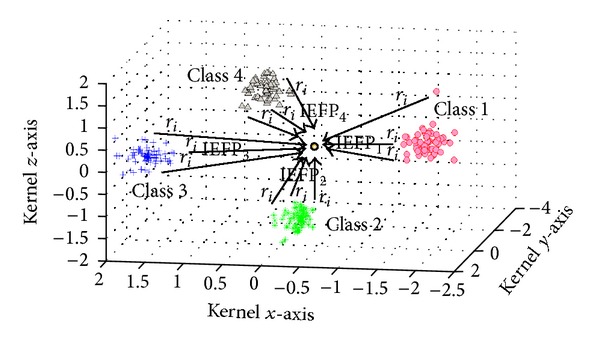
IEFP with different classes in 3D kernel space.

Therefore, in class intensity model, IEFP is a criterion of classification. For example, there are four classes in training data: class 1, class 2, class 3, and class 4 in Figure 3. After projecting with kernel methods, all projection class charge points of training data can form a space electric field. The test sample can be projected onto this space with the same kernel methods. Figure 3 illustrates the relationship between point charge of different class and corresponding IEFP. To project position of test sample, if there exist IEFP_1_ > IEFP_2_,  IEFP_1_ > IEFP_3_ and IEFP_1_ > IEFP_4_, test sample should belong to class 1.

## 3. Results and Discussion

### 3.1. System and Software Used for Data Analysis

The calculations were carried out using the Intel(R) Core(TM) Duo CPU T5870 GHz computer running Windows XP operating system. All the learning input data were range-scaled to [0~1] in this work. The improved 3D kernel approach software package including 3D kernel PCA and 3D GDA was programmed in our laboratory referring to the literature [29, 31] based on statistical pattern recognition toolbox for MATLAB [35]. 

### 3.2. Application of Improved 3D Kernel Approach to Protein's Tertiary Structure Classes of Domains

The protein datasets studied here were taken from Niu and his coworkers [17]. In dataset A, there are 277 protein domains, of which 70 are all-*α* domains, 61 all-*β*, 81 *α*/*β*, and 65 *α* + *β*. In dataset B, there are 498 protein domains, of which 107 are all-*α* domains, 126 all-*β*, 136 *α*/*β*, and 129 *α* + *β*. The amino acid composition was used to represent the sample of a protein domain.

To demonstrate the power of 3D kernel methods, computations were performed by the Leave-One-Out Cross-Validation (LOOCV), which are widely used by more and more investigators in testing the power of various predictors. As such, the data set of *n* samples was divided into two disjoint subsets including a training data set (*n* − 1 samples) and a test data set (only 1 sample). After developing each model based on the training set, the omitted data was predicted and the difference between experimental value and predicted value was calculated [36–38]. 

Based on dataset A, it was found that the projection with Gaussian (see (3), *σ* = 0.5) kernel function and KNN (*K* = 3) algorithm estimation was suitable for building 3D kernel PCA model with the better success rates.

**Figure 4:**
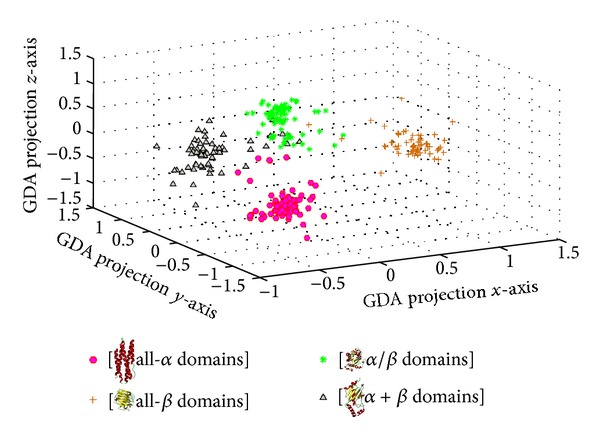
Distribution of different protein's tertiary structure classes data in 3D kernel space.

Based on dataset B, it was found that the projection with polynomial (see (2), *d* = 4, *θ* = 1.5) kernel function and class intensity model estimation was suitable for building 3D GDA model with the better success rates.  Figure 4 illustrates the protein domains classes distribution of dataset B (498 samples) in 3D kernel space with GDA model. It can be seen that the data points, which belong to all-*α* domains, all-*β* domains, *α*/*β* domains, and *α* + *β* domains respectively, are located in different regions with a correct classification result. 

The success rates thus obtained are given in Table 1, where, for facilitating comparison, the corresponding rates obtained by component-coupled algorithm, neural networks, support vector machines (SVMs), and AdaBoost Learner [17] are also listed.

As it can be seen from Table 1, the performance of improved 3D kernel model outperforms those of component-coupled, neural networks, SVMs models but was a little worse than that of AdaBoost model for the dataset A (277 domains) available in LOOCV test. Based on dataset B (498 domains), improved 3D kernel learner is superior to all the other predictors in identifying the structural classification.

### 3.3. Application of Improved 3D Kernel Approach to Classification of Membrane Proteins

**Figure 5:**
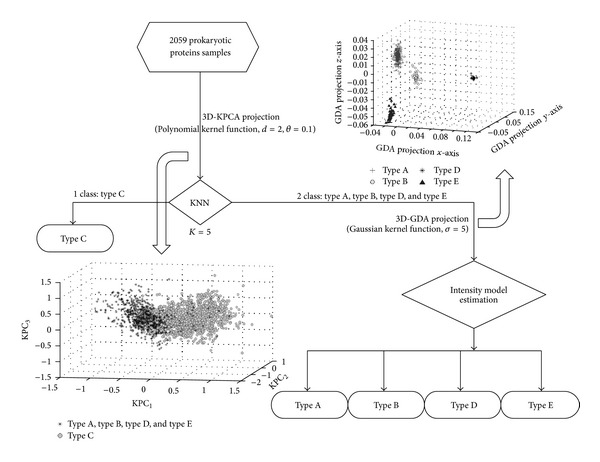
Classification flow chart of five type membrane proteins.

The membrane proteins dataset studied here was collected from the literature [25]. The dataset contains 2059 prokaryotic proteins (type A membrane proteins: 435; type B membrane proteins: 152; type C Multi-pass transmembrane proteins: 1311; type D lipid chain-anchored membrane proteins: 51; type E GPI-anchored membrane proteins: 110). The amino acid composition was selected as the input of the classification algorithm, and the computations were performed by LOOCV to test the power of various predictors. Based on dataset of membrane proteins, the classification flow chart (Figure 5) was obtained as follows. 

**Figure 6:**
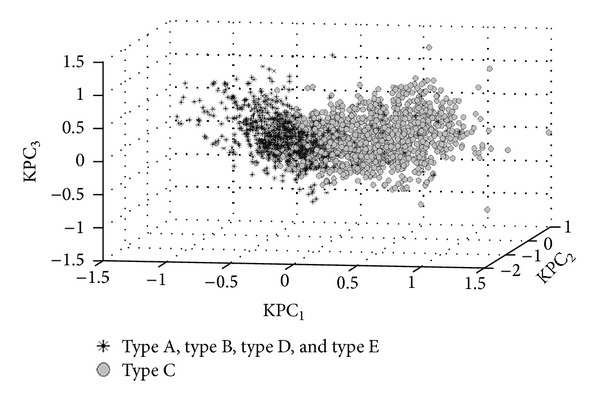
Distribution of the multipass transmembrane proteins (type C) and the other membrane proteins (type A, type B, type D and type E) data in 3D kernel space.

From Figure 5, there are two steps in building classification model. Firstly, the 3D KPCA model with projection through polynomial (see (2), *d* = 2, *θ* = 0.1) kernel function and KNN (*K* = 5) algorithm estimation was built to classify the multipass transmembrane proteins (type C) and the other membrane proteins (type A, type B, type D, and type E).  Figure 6 illustrates the data distribution of type C and other membrane proteins in 3D kernel space with KPCA model. 

Secondly, the 3D GDA model with Gaussian (see (3), *σ* = 5) kernel function and class intensity model estimation was built to classify type A, type B, type D, and type E membrane proteins. 

**Figure 7:**
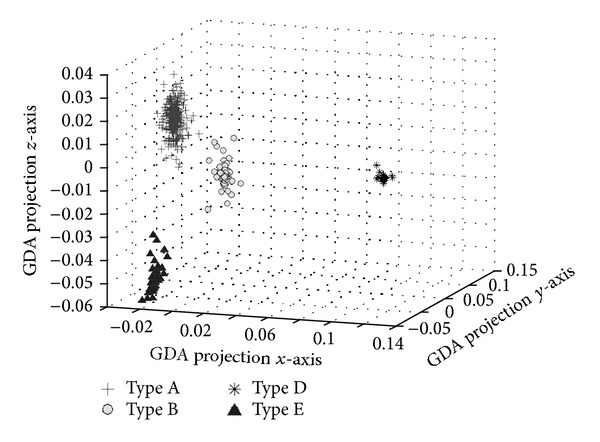
Distribution of the type A, type B, type D, and type E data in 3D kernel space.

Figure 7 illustrates the data distribution of the type A, type B, type D, and type E membrane proteins in 3D kernel space with GDA model. 3D kernel method was compared with other machine learning classification methods: the covariant discriminant algorithm [23], neural networks, support vector machines, and Bagging [25], as is shown in Table 2. 

As we can see from Table 2, correct classification rate of the LOOCV test applied 3D kernel algorithm outperformed other algorithms. It also means that 3D kernel method has learned very well through the membrane proteins training process. 

## 4. Conclusions

The 3D kernel approach is very useful machine learning classifier. It has remarkably outperformed the powerful neural network, SVM classifiers, in predicting the protein domain structural classes for the two datasets constructed and membrane protein types for the same dataset constructed by previous investigators. It is thus anticipated that the 3D Kernel classifier can also be used to predict other protein attributes, such as sub-cellular localization [39–41], enzyme family and subfamily classes [42], and active sites of enzyme. The concepts of EQPC and IEFP can be easily extended to many-dimensional space and could be improved to use four or more dimensions. 

It could be concluded that 3D kernel approach is a robust and highly accurate classification technique that can be successfully applied to derive statistical models with statistical qualities and predictive capabilities for the protein location and function. The 3D kernel algorithm should be a complementary tool to the existing pattern recognition in chemometrics and bioinformatics. 

## Figures and Tables

**Table 1:** LOOCV success rates by component-coupled, neural network, SVMs, AdaBoost, and improved 3D kernel approach.

Dataset	Algorithm	All-*α*	All-*β*	*α*/*β*	*α* + *β*	Overall
Dataset A (277 domains)	Component-coupled	84.3%	82.0%	81.5%	67.7%	79.1%
	Neural networks	68.6%	85.2%	86.4%	56.9%	74.7%
	SVMs	74.3%	82.0%	87.7%	72.3%	79.4%
	AdaBoost	87.1%	95.1%	98.7%	81.5%	90.9%
	3D kernel	88.6%	85.3%	93.8%	77.0%	86.6%

Dataset B (498 domains)	Component-coupled	93.5%	88.9%	90.4%	84.5%	89.2%
	Neural networks	86.0%	96.0%	88.2%	86.0%	89.2%
	SVMs	88.8%	95.2%	96.3%	91.5%	93.2%
	AdaBoost	96.2%	92.1%	98.5%	89.9%	94.2%
	3D kernel	91.6%	95.3%	99.3%	92.3%	95.0%

**Table 2:** LOOCV success rates by covariant discriminant, neural network, SVM, bagging, and improved 3D kernel approach.

Algorithm	Rate of correct prediction for each class					Overall rate of correct prediction
	Type A	Type B	Type C	Type D	Type E	
Covariant discriminant	74.0%	52.0%	83.7%	49%	45.4%	76.4%
Neural network	75.63%	30.92%	88.86%	50.98%	30.91%	77.76%
SVMs	77.7%	28.3%	92.5%	52.9%	35.5%	80.9%
Bagging	79.80%	48.68%	93.21%	49.02%	60.91%	84.18%
3D kernel	78.11%	31.02%	94.36%	52.63%	45.46%	84.50%
